# Spatio-temporal Integration of Speech Reflections in Hearing-Impaired Listeners

**DOI:** 10.1177/23312165221143901

**Published:** 2022-12-19

**Authors:** Jan Rennies, Anna Warzybok, Birger Kollmeier, Thomas Brand

**Affiliations:** 1 28439 Fraunhofer Institute for Digital Media Technology IDMT, Project Group Hearing, Speech and Audio Technology, Oldenburg, Germany; 2 Medical Physics Group, Department für Medizinische Physik und Akustik, Oldenburg, Germany; 3 Cluster of Excellence Hearing4all, Oldenburg, Germany

**Keywords:** hearing impairment, speech intelligibility, binaural hearing, temporal integration, reflections

## Abstract

Speech recognition in rooms requires the temporal integration of reflections which arrive with a certain delay after the direct sound. It is commonly assumed that there is a certain temporal window of about 50–100 ms, during which reflections can be integrated with the direct sound, while later reflections are detrimental to speech intelligibility. This concept was challenged in a recent study by employing binaural room impulse responses (RIRs) with systematically varied interaural phase differences (IPDs) and amplitude of the direct sound and a variable number of reflections delayed by up to 200 ms. When amplitude or IPD favored late RIR components, normal-hearing (NH) listeners appeared to be capable of focusing on these components rather than on the precedent direct sound, which contrasted with the common concept of considering early RIR components as useful and late components as detrimental. The present study investigated speech intelligibility in the same conditions in hearing-impaired (HI) listeners. The data indicate that HI listeners were generally less able to “ignore” the direct sound than NH listeners, when the most useful information was confined to late RIR components. Some HI listeners showed a remarkable inability to integrate across multiple reflections and to optimally “shift” their temporal integration window, which was quite dissimilar to NH listeners. This effect was most pronounced in conditions requiring spatial and temporal integration and could provide new challenges for individual prediction models of binaural speech intelligibility.

## Introduction

Speech signals arriving at the listener’s ears in real listening scenarios consist of the direct sound, the “early” reflections within the first 50–100 ms after the direct sound, as well as later reflections that ultimately fuse to the late reverberation. It is generally agreed that early reflections are beneficial, that is, that they can be integrated (at least partially) with the direct sound causing an improvement of speech recognition (e.g., Lochner & Burger, 1964; Bradley et al., 2003; Arweiler & Buchholz, 2011; Warzybok et al., 2013). In contrast, late reflections cannot be integrated with the direct sound and can be detrimental for speech intelligibility (e.g., Steeneken & Houtgast, 1980; George et al., 2010; Rennies et al., 2011; Hochmuth et al., 2015). The concept of separating early (assumed useful) and late (assumed detrimental) reflections is the basis for standard room acoustical measures such as the clarity or definition measure. In a previous study (Rennies et al., 2019), we investigated how the integration of speech reflections depended on their amplitude, their delay relative to the direct sound as well as on the binaural information contained in them (such as the interaural phase differences, IPDs) in listeners with normal hearing. In this article, we extend this study by testing a group of listeners with sensorineural hearing loss.

Many studies have investigated the temporal integration of speech reflections and reverberation. For very short reflection delays, some studies found the same improvement in speech intelligibility irrespective of whether the speech energy was added as reflection(s) or to the direct sound, which indicates that the reflection could be perfectly integrated with the direct sound. The temporal window for perfect integration was reported to be between 25 and 50 ms (Lochner & Burger, 1964; Nábělek & Robinette, 1978; Bradley et al., 2003; Warzybok et al., 2013; Leclère et al., 2015; Rennies et al., 2019). In contrast, other studies found that adding speech energy as early reflections was less beneficial than adding the same energy as direct sound (Parizet & Polack, 1992; Soulodre et al., 1989; Arweiler & Buchholz, 2011), that is, the temporal integration of early reflections was less than perfect even at very short delays. This would also be in line with predictions of the Speech Transmission Index (STI). The STI is based on the average of the modulation transfer function across modulation frequencies, which continuously decreases as the delay of a single reflection increases (IEC, 2003; Rennies et al., 2014; Roettges et al., 2022).

Only few studies explicitly investigated the interaction between temporal integration of speech reflections and binaural processing. Arweiler & Buchholz (2011) measured speech recognition thresholds (SRTs, i.e., the signal-to-noise ratios (SNRs) required to achieve 50% speech intelligibility) in diffuse noise. They varied the SNR by either increasing the direct sound energy or the energy of 20 early reflections (all within 55 ms after the direct sound), which were presented from their original azimuth and elevation (the direct sound was presented from the front). As control conditions, Arweiler & Buchholz (2011) also presented all reflections from the frontal loudspeaker (i.e., co-located with the direct sound) and, in addition, included monaural presentation. The main findings were that an increase in direct-speech energy was more beneficial than an equivalent increase in reflection energy, and that this difference was smaller when all reflections were presented from the front. Arweiler & Buchholz (2011) concluded that temporal integration of early reflections was facilitated when they arrived from the same direction as the direct sound. In addition, they found that SRTs were lower in binaural than in monaural listening conditions due to spatial unmasking in the presence of the diffuse masker. This SRT benefit was the same for frontal and spatially distributed early reflections and, hence, Arweiler & Buchholz (2011) argued that the binaural system could not integrate early reflections more efficiently than the monaural system and that, therefore, temporal and binaural processing was independent.

Warzybok et al. (2013) confirmed this finding for conditions with frontal direct sound and a single, equally strong frontal reflection. In comparison to a reference condition with co-located noise, they found SRTs to decrease by a constant amount of about 4 and 8 dB when using diffuse noise or lateral noise, respectively, independently of the delay of the reflection. However, this was no longer the case when the reflection was not co-located with the direct sound: When the reflection delay was short (≤50 ms), SRTs were the same as for co-located reflections. In contrast, when a long delay of 200 ms was used, the detrimental effect observed for co-located reflections was considerably reduced when the reflection arrived from the same hemisphere as the lateral noise, but not when it arrived from the opposite hemisphere. This was called “suppression effect” of a detrimental reflection by Warzybok et al. (2013) and was interpreted as an indication for an interaction of binaural and temporal processing. Rennies et al. (2013) showed that this was also the case for listeners with sensorineural hearing loss. Recently, Rennies et al. (2019) further extended the paradigm of Warzybok et al. (2013). They used artificial binaural room impulse responses (BRIRs) and systematically manipulated the reflection delay, reflection amplitude, number of reflections as well as the binaural information contained in individual components of the BRIR (by manipulating the IPD). They found that the direct sound and one or several speech reflections could be perfectly integrated when they had the same IPD and that, in such conditions, temporal and spatial processing were independent from each other. In contrast, a single or small number of early reflections with the same IPD as the noise (but not as the direct sound) could not be perfectly integrated with the direct sound even at short reflection delays. These results were in agreement with Warzybok et al. (2013), and could be modeled with a classic approach of binaural processing in combination with a separation of the BRIR into an early (assumed as useful) and a late (assumed as detrimental) part (Rennies et al., 2014, 2019; Leclère et al., 2015). This model approach (like the definition and clarity measures) is based on the assumption that the useful part consists of the early BRIR components (i.e., direct sound plus reflections up to a certain delay), while the detrimental part consists of the later reflections (including late reverberation). This concept was challenged by Rennies et al. (2019) by creating conditions in which the presumably dominant source of information was confined to late BRIR components, which are typically considered detrimental. This was achieved by increasing the energy of late reflections relative to the direct sound and (more interestingly) by providing an IPD cue relative to the masking noise only in late reflections, but not in the direct sound or the early reflections. With convincing consistency across a series of experiments, Rennies et al. (2019) found that the normal auditory system appears to be capable of focusing on these late components rather than on the direct sound and the subsequent early components whenever energy and/or binaural information make them a more dominant cue for speech understanding. In other words, the late BRIR components “took over” the role of the primary source of information from the preceding direct sound regardless of their delay, while the direct sound could be ignored if that provided an advantage. An IPD advantage seemed to dominate speech intelligibility also when the level of the direct sound and the reflection were the same. In such conditions the late reflection would produce an increase in SRTs at each ear considered separately. This finding could not be predicted by the classic useful/detrimental separation approach. Instead, a more flexible model stage was required for temporal integration, which allowed the model to use the useful reflections regardless of their temporal position in the BRIR, that is, even if they occurred outside the classic early window (Rennies et al., 2019). This model approach could be interpreted as a generalization of the useful/detrimental separation.

The present study explored if and to what degree these effects are also present in listeners with hearing loss. If hearing impairment would simply affect the level and spectral shape of the internally perceived sound by these listeners, we would expect no difference to the findings in normal-hearing (NH) listeners if an appropriate frequency-dependent amplification is applied. However, even above threshold and/or after appropriate amplification, it is well known that sensorineural hearing loss affects binaural processing capabilities resulting in, forexample, reduced sensitivity to interaural time differences (e.g., Neher et al., 2011; King et al., 2014; Füllgrabe & Moore, 2017), binaural pitch perception (e.g., Santurette & Dau, 2012), binaural masking level differences (e.g., Hall & Harvey, 1985), or binaural intelligibility level differences (e.g., Goverts & Houtgast, 2010). Some authors attribute this specific binaural impairment to a separate factor in modelling individual effects of speech intelligibility (e.g., Kollmeier & Kiessling, 2018) and found (at least slightly) improved prediction accuracy when including individually adjusted binaural processing errors in their models (e.g., Brand et al., 2018, also in Vicente et al., 2021, to account for level-dependent effects of binaural unmasking). In contrast, the majority of other studies reported good prediction accuracy of binaural speech intelligibility for hearing-impaired (HI) listeners without including a specific binaural impairment factor (e.g., Beutelmann et al., 2010; Schädler et al., 2018; Kubiak et al., 2020; Vicente et al., 2020; Lavandier et al., 2021). Hence, a direct comparison between the performance of NH and HI listeners with a sensorineural hearing loss in an ecologically relevant task, that is, speech recognition in a noisy and reverberant environment, could shed some light on the yet unresolved issue of a specific binaural hearing impairment. To this end, a group of HI listeners was measured under spatio-temporal conditions that exactly matched those tested in our earlier study with NH listeners. This allowed us to address the following research questions:

A: Can the tested HI listeners make use of early reflections with or without additional binaural information in the same way as NH listeners?
B: Are they also capable of “ignoring” the direct sound if the late parts of the BRIR contain more relevant information due to their energy or IPD cues? And can they flexibly “shift” their temporal integration window as observed for NH listeners?
C: Can the tested HI listeners cope with having to integrate across multiple reflections?
D: Can interindividual variability in performance in complex spatio-temporal conditions be predicted by average hearing loss, low-frequency hearing thresholds (assumed most relevant for IPD-processing), or general speech recognition performance in less complex conditions for the present group of HI listeners?

## Methods

### Listeners

**Figure 1.**
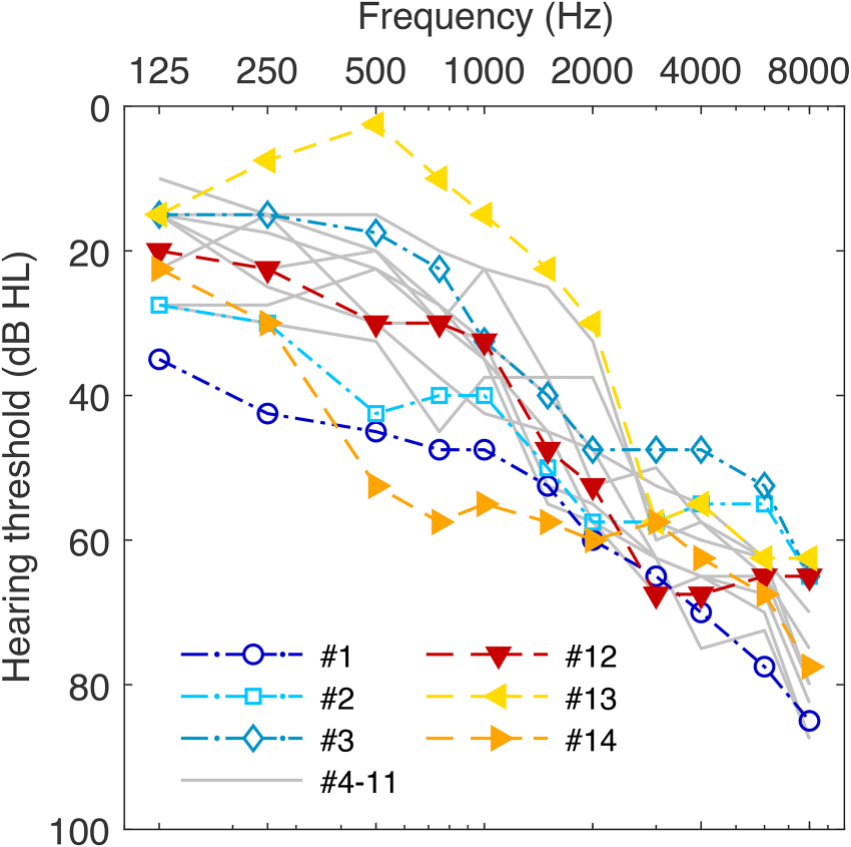
Average Air-Conduction Audiograms Across Ears for all 14 Listeners. Each line represents one listener. The three listeners with the worst speech recognition thresholds (SRTs) in the reference condition (#1–3) are indicated by open symbols with dash-dotted lines; the three best listeners (#12–14) by filled triangles and dashed lines. The remaining listeners are indicated by gray solid lines.

Fourteen native German listeners (five female, nine male) between 45 and 84 years of age participated in this study. The majority was older than 70 years at the time of their participation. Their audiometric details are summarized in Table 1, and average air-conduction audiograms across ears are shown in Figure 1. All listeners had post-lingual, mild to moderate, symmetrical, sloping, hearing loss with differences in pure-tone average (PTA${}_{4}$, i.e., the average of hearing levels at 0.5, 1, 2, and 4 kHz) of no more than 11 dB between both ears. Their hearing thresholds were also symmetrical in the lower frequency region up to 1 kHz: the average hearing levels across audiometric frequencies up to 1 kHz (referred to as PTA${}_{\mathrm{LF}}$ in the following) differed by less than 9 dB across ears for all subjects, although differences at individual frequencies of 15 or 20 dB were observed for four listeners, see Table 1. Average PTA${}_{4}$ and PTA${}_{\mathrm{LF}}$ across ears ranged from 26 to 58 dB HL and from 10 to 44 dB HL, respectively, that is, some listeners’ hearing thresholds were within the normal range at low frequencies. Hearing losses were primarily of sensorineural origin, as average air-bone gaps (ABGs) across ears were less than 9 dB for all but one subject (#4, see Table 1). The etiology of the subjects’ hearing loss was not known and was not diagnosed in this study. In relation to their age, the hearing loss of our listeners was mostly more pronounced than would be expected based on their age alone according to ISO (2013), which is applicable to the highly screened, otologically normal population. The extreme case was listener #14 (45 years) with a difference between actual (53.8 dB HL) and age-expected PTA${}_{4}$ (3.5 dB HL) of more than 50 dB. During the SRT measurements of this study, reduced audibility was partly compensated for by applying individualized linear gain according to the NAL-RP prescription rule (Dillon, 2012) before stimulus presentation. The aim of the NAL-RP rule is to amplify the sound spectrum to be audible while avoiding too loud signals in the present experimental setup. Note that the hearing loss was too large for the amplification rule to restore audibility at high audiometric frequencies, that is, the sensation levels were lower than 0 dB SL for frequencies above between 4 and 6 kHz, depending on the individual hearing loss. Listeners conducted the measurements in two sessions of 1–2 h, were paid for their participation, and gave an informed consent. All procedures were approved by the ethics committee of the University of Oldenburg (Protocol Drs.EK/2019/022).

**Table 1.** Air-Conduction Audiograms for Left (L) and Right (R) Ears of all Listeners Together With PTA${}_{4}$, PTA${}_{\mathrm{LF}}$, and Air-Bone Gap (ABG). The Last Column Indicates the Number of Adaptive SRT Tracks, Which Could not be Completed (See Text).

#	Age	Air-conduction hearing thresholds (dB HL) at frequency (kHz)												PTA${}_{4}$	PTA${}_{\mathrm{LF}}$	ABG	# Aborted
	(y)		0.125	0.25	0.5	0.75	1	1.5	2	3	4	6	8	(dB HL)	(dB HL)	(dB)	tracks
1	79	L	35	40	50	50	45	50	55	60	65	75	85	53.8	44.0	3.6	3
		R	35	45	40	45	50	55	65	70	75	80	85	57.5	43.0	2.9	
2	76	L	25	30	40	30	35	60	65	70	55	65	70	48.8	32.0	0.7	3
		R	30	30	45	50	45	40	50	45	55	45	60	48.8	40.0	2.9	
3	77	L	15	15	20	20	30	40	50	50	50	55	60	37.5	20.0	2.9	2
		R	15	15	15	25	35	40	45	45	45	50	70	35.0	21.0	0.0	
4	50	L	30	30	25	25	30	50	55	65	65	70	85	43.8	28.0	13.6	2
		R	25	25	20	30	35	55	55	60	65	70	90	47.5	43.8	27.0	
5	84	L	25	20	15	20	25	35	35	60	55	60	75	32.5	21.0	3.6	–
		R	20	10	15	20	20	40	40	55	65	65	85	35.0	17.0	5.7	
6	83	L	10	15	20	35	35	45	50	55	55	60	70	40.0	23.0	1.4	2
		R	20	20	25	25	30	35	45	50	55	65	70	38.8	24.0	0.7	
7	77	L	20	25	30	40	35	35	50	50	55	55	75	42.5	30.0	3.6	–
		R	35	35	35	50	40	40	55	50	60	70	85	47.5	39.0	7.1	
8	76	L	15	25	20	30	40	50	55	75	85	80	95	50.0	26.0	5.0	–
		R	15	20	20	30	30	40	40	50	65	65	80	38.8	23.0	5.0	
9	81	L	10	10	25	35	40	40	55	60	65	65	75	46.3	24.0	1.4	2
		R	20	20	35	40	45	50	60	65	65	70	90	51.3	32.0	1.4	
10	79	L	10	15	20	25	30	55	65	70	70	70	85	46.3	20.0	11.4	1
		R	10	15	20	30	40	55	50	65	60	60	75	42.5	23.0	5.7	
11	82	L	15	25	30	30	20	25	35	60	60	75	100	36.3	24.0	3.6	1
		R	15	25	30	30	25	25	30	60	55	55	50	35.0	25.0	3.6	
12	81	L	20	25	30	25	25	40	50	65	70	65	65	43.8	25.0	6.4	–
		R	20	20	30	35	40	55	55	70	65	65	65	47.5	29.0	6.4	
13	73	L	15	5	0	5	10	25	35	55	50	60	60	23.8	7.0	1.4	–
		R	15	10	5	15	20	20	25	60	60	65	65	27.5	13.0	2.1	
14	45	L	20	25	45	55	50	60	60	60	60	65	75	53.8	39.0	5.0	–
		R	25	35	60	60	60	55	60	55	65	70	80	61.3	48.0	4.3	

### Stimuli and Conditions

As in the previous study with NH listeners, SRTs were measured using a matrix sentence test. The target speech was uttered by a male talker and consisted of sentences taken from the Oldenburg sentence test (Wagener et al., 1999), for example, *“Peter kauft acht nasse Steine.”* [*“Peter buys eight wet stones”*]. These sentences always have the fixed five-word structure name-verb-numeral-adjective-object. For each word group ten alternatives are available, which can be randomly combined to produce syntactically correct, but semantically unpredictable sentences. The test material consists of 100 such sentences, which are combined with lists of 20 sentences. The sentences as well as their combinations to obtain lists have been optimized to produce highly homogeneous SRTs (see Wagener et al., 1999). Due to the lack of semantic predictability, memorizing any of the 100 sentences is not likely (i.e., each sentence appears to the listeners as one of the 10${}^{5}$ possible random combinations), allowing for multiple measurements with the same target material. The masker consisted of stationary speech-shaped noise generated from the speech material so that the long-term noise spectrum matched that of the speech material. This type of matrix tests exists in various languages, with the same design principle, test-specific noise, optimization and evaluation procedures in each language (Kollmeier et al., 2015). Hence, measurements in the same acoustic conditions should be highly comparable across languages, although each test has its own (native) target talker, and offsets in SRT and slope have to be taken into account, which may be due to talker-specific effects (see Figure 6 in Kollmeier et al., 2015). To be able to quantitatively compare the data of (German) HI listeners collected in the present study with data of (US American) NH listeners of our previous study (Rennies et al., 2019), the expected SRT offset between the two matrix tests was calculated. This was possible because the reference condition (*D*${}_{0}$*N*${}_{0}$, see below) of this study and the previous study with American English NH listeners had also been measured with German NH listeners by Warzybok et al. (2013) using exactly the same speech material as in the present study. Rennies et al. (2019) distributed their various conditions across two different groups of NH listeners (each group measured the reference condition). Reference SRTs of these two groups were lower in English natives by 4.3 and 4.8 dB, respectively, compared to the reference SRT measured for German natives by Warzybok et al. (2013). This difference in reference SRT across languages is slightly larger than the 2.9 dB expected from the reference SRT data (Kollmeier et al., 2015) and is mainly due to speaker-specific effects, to a much smaller degree due to the structure of the specific language (Hochmuth et al., 2015), and likely also due to differences in listener panels. For the analyses of the present study, these values were added to the corresponding NH data shown below in order to quantitatively compare the data of HI and NH listeners for the same conditions. This cross-language comparison was necessary since, apart from the reference condition, no NH reference data were available for the conditions under test.

The desired combinations of speech components (direct sound, *D*, and one or several reflections, *R*) and noise (*N*) were generated exactly as in our previous study, that is, the target speech was convolved with artificial binaural room impulse responses (BRIRs). The BRIRs were created based on the BRIR for frontal sound incidence in anechoic conditions (i.e., without reflections) employed by Warzybok et al. (2013). This BRIR had been simulated with the CATT Acoustic software v8.0a (CATT, Gothenburg, Sweden) by using an omnidirectional source in an anechoic room and modeling the receiver as a head-and-torso simulator (KEMAR; G.R.A.S., Sound & Vibration, Holte, Denmark) at a distance of 5 m from the source. This BRIR comprising only direct sound was used as the basic component to create new BRIRs by introducing identical copies at specific delays to produce the desired reflections, resulting in BRIRs with between 1 (direct sound only) and 10 components (direct sound plus nine reflections). The delay Δ*t* of the reflections was varied systematically, and was 10, 25, 50, 75, 100, 125, 150, 175, and/or 200 ms. In addition, the IPD of the different components was manipulated, that is, the direct sound was either diotic (*D*${}_{0}$) or had an IPD of $180^{\circ }$ (*D*${}_{\pi }$). The IPD manipulation was realized by inverting the phase at the left ear. The same IPD manipulation was conducted for some or all of the individual reflections (denoted by *R*${}_{0}$ or *R*${}_{\pi }$ in the following) and the masking noise (*N*${}_{0}$ or *N*${}_{\pi }$). In most experiments of this study, all components of the BRIR had the same level, but in some experiments the relative levels of direct sound and reflection were varied. This was achieved by multiplying the reflection by an amplitude amplification factor α before copying the BRIR component at the desired delay of the BRIR. The amplification factor was always applied to both ears in the same way. This manipulation ensured that both speech and noise always had the same level at both ears and, hence, that there was no monaural SNR advantage at either ear.

Note that, as in the previous study, the overall speech level was always calculated including all speech components. This means that, for speech stimuli with at least one reflection, the absolute level of the direct sound was reduced at a given overall speech level, because the energy was spread across the BRIR components. This reduction of the direct sound level depended on reflection amplitude and the number of reflections (cf. Table 2 in Rennies et al., 2019). For example, when a single reflection with the same amplitude as the direct sound was added to the direct sound (as in Exp. I to III), each component was decreased by about 3 dB to obtain the same overall speech level. For more than one reflection added (as in Exp. VI to IX), the level of each component decreased accordingly by up to about 10 dB for the largest number of reflections employed in this study.

The present study comprised a subset of the conditions measured by Rennies et al. (2019). Altogether, 22 different conditions were created with different parameter variations. For the sake of clarity, these conditions are combined and discussed as nine experiments below, where some conditions served as anchor points for several experiments (e.g., the “direct sound-only” condition and the conditions with nine reflections were included as reference points in several experiments as described in the following, that is, some data points are re-plotted in the several experiments). During the measurements, all conditions were pooled and randomized for each subject (see below). Table 2 summarizes the different combinations of BRIR components, the reflection delays relative to the direct sound, their amplitude amplification factor α, their IPD, and the IPD of the noise. In Exp. I to V, the target speech always consisted of the direct sound and a single reflection, and the IPDs (of *D*, *R*, and *N*), the reflection delay as well as the reflection amplification factor α were varied. Exp. I comprised baseline measurements of binaural processing (no temporal integration), while Exp. II comprised a baseline for temporal integration (no binaural processing). Exp. III to V tested simultaneous temporal integration and binaural processing. In Exp. IV and V, the relative amplitude of the reflection was varied in diotic (IV) and binaural (V) conditions. Exp. VI to IX comprised BRIRs with several reflections. In Exp. VI and VII, the effect of adding an increasing number of reflections was investigated, and the reflections were successively added starting from the lowest delay (i.e., at 10 ms and then increasing). Exp. VIII and IX also explored the effect of adding an increasing number of reflections, but here the reflections were successively added from the largest delay (i.e., at 200 ms and then decreasing).

**Table 2.** Overview of Measurement Conditions. The Second and Third Columns Indicate the IPD of the Direct Sound (*D*) and Noise (*N*), Respectively, While the Remaining Columns Indicate the Properties of the Reflection(s).

Exp.	D-IPD	N-IPD	refl.	Reflection delay	Reflection	R-IPD
				Δ*t* (ms)	amp. α	
I	0	0	0	–	–	–
	0	π	0	–	–	–
	π	0	0	–	–	–
II	0	0	1	10, 100, 200	1	0
III	*– Same as Exp. II, but with R${}_{\pi }$ instead of R${}_{0}$ –*					
IV	0	0	1	200	0, 0.75, 1.0,	0
					1.25, 2.5	
V	*– Same as Exp. IV, but with R${}_{\pi }$ instead of R${}_{0}$ –*					
VI	0	0	1	10	1	0
			5	10, 25, 50, 75, 100		
			9	10, 25, 50, 75, 100,		
				125, 150, 175, 200		
VII	*– Same as Exp. VI, but with R${}_{\pi }$ instead of R${}_{0}$ –*					
VIII	0	0	1	200	1	0
			5	200, 175, 150,		
				125, 100		
			9	200, 175, 150, 125,		
				100, 75, 50, 25, 10		
IX	*– Same as Exp. VIII, but with R${}_{\pi }$ instead of R${}_{0}$ –*					

### Calibration and Equipment

The masker level was always fixed at a 65 dB sound pressure level (SPL, as in Warzybok et al., 2013; Rennies et al., 2019) and the speech level was adjusted to converge to the SRT. All stimuli were generated and controlled using Matlab (Natick, MA, USA). The AFC-Matlab framework of Ewert (2013) was used to measure SRTs. The digital output was D/A converted via an RME Fireface UC (ASIO) sound card (RME, Chemnitz, Germany), amplified using a Tucker-Davis HB7 headphone amplifier (Tucker-Davis Technologies, Alachua, FL, USA), and delivered to the listeners via HD650 headphones (Sennheiser, Wedemark, Germany) in a sound-attenuated booth. The setup was calibrated for SPL using a Brüel and Kjær (B&K, Nærum, Denmark) 4153 artificial ear, a B&K 4143 1/2 in. microphone, a B&K 2669 preamplifier, and a B&K 2610 measuring amplifier. The right ear served as a reference point for the calibration, but the level of the two ears was always the same. The actual output levels differed for each subject due to the individual NAL-RP amplification.

### Procedure

The SRT measurements were conducted using an open-set response format, that is, listeners repeated the words they had recognized after each sentence and an experimenter marked with the correct words on a graphical user interface (not visible for the subjects), then the next sentence was played. This was contrary to the closed-set response format employed by Rennies et al. (2019) in which the listeners selected the recognized words on a touchscreen displaying the entire 50-word matrix. The open-set format was chosen because, in our experience, this is less tiring for elderly listeners. Two initial training SRTs were measured using lists of 20 sentences of the original matrix test (i.e., without BRIR convolution) to familiarize the listeners with the speech material and the task to reduce training effects typical for matrix sentence test (Kollmeier et al., 2015). Subsequently, the experimental conditions were measured, each with a new random list of 20 sentences. The initial SNR of each adaptive track was 0 dB. The SNRs of the subsequent sentence presentations varied adaptively, that is, the step size of the speech level depended on the number of correctly understood words and decreased exponentially after each reversal of the presentation level using the procedure described by Brand & Kollmeier (2002) to converge to the SRT. The maximum initial step size in case all five words of the first sentence were recognized correctly or incorrectly was −7.5 dB and +7.5 dB, respectively. The final SRT estimate of each test list was obtained by fitting a sigmoidal function (with 0% guess rate due to the open response format) to the measured data and deriving the SRT from this function. The maximum allowed SNR of the adaptive procedure was +20 dB to avoid too high output levels (i.e., more than 85 dB SPL plus individual NAL-RP amplification). The procedure was implemented to abort if this SNR limit was hit more than three times. This had never happened during the measurements with NH listeners, but did occur for some listeners and conditions in the present study as reported below. All conditions of all experiments were pooled and randomized. The SRT for each condition was measured completely before proceeding to the next condition. SRTs were tested for normality using Shapiro-Wilk tests and analyzed by means of repeated-measures analyses of variance (ANOVA), including Greenhouse-Geisser corrections of degrees of freedom. *t*-Tests were conducted as post-hoc tests, and Bonferroni-corrected for multiple comparisons.

## Results and Discussion

### Experiment I: Baseline Performance

**Figure 2.**
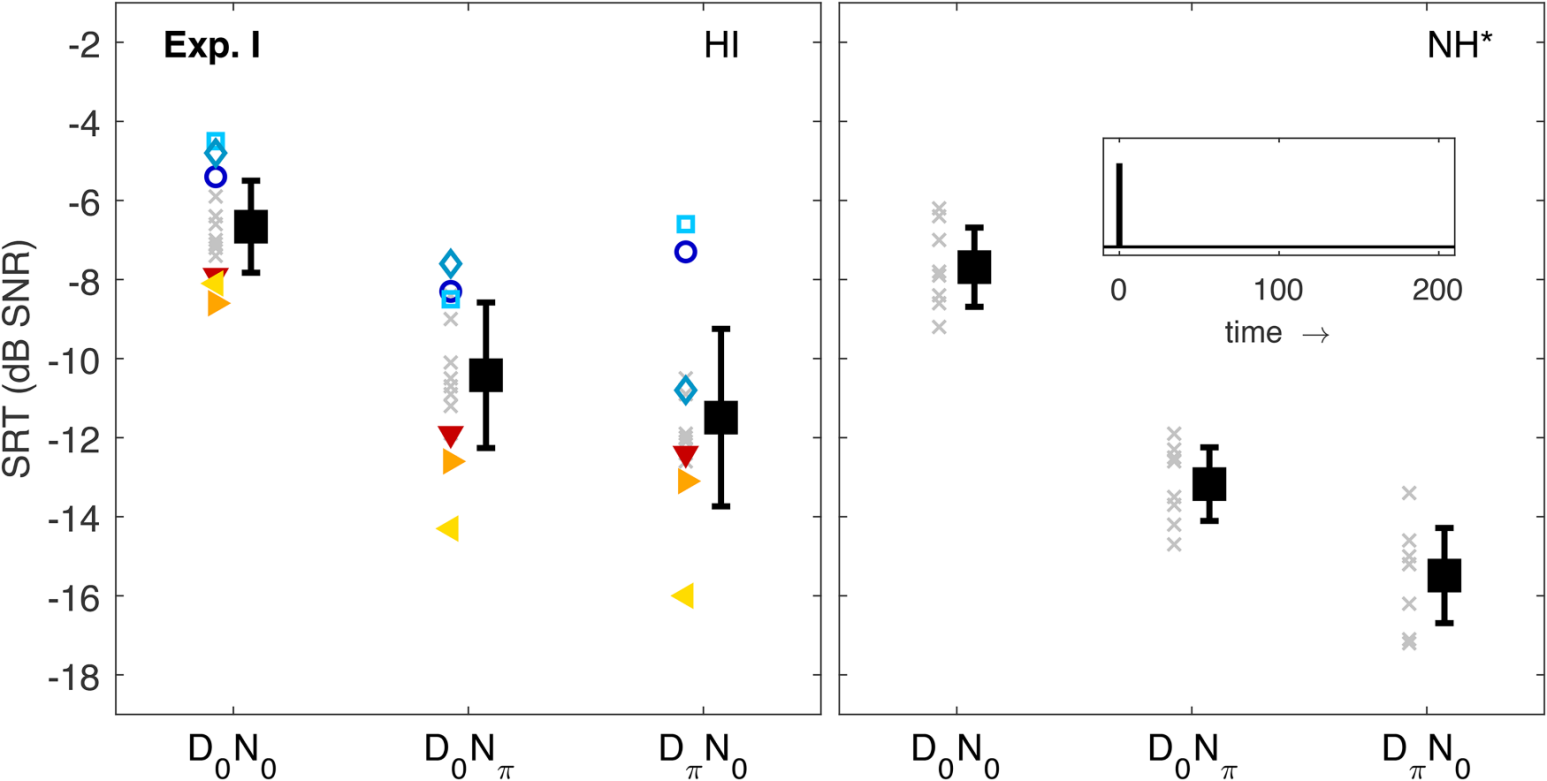
SRTs of Exp. I (left panel). Black squares and error bars indicate the interindividual mean values and standard deviations. The small symbols indicate individual data. The three listeners with the worst SRTs in the reference conditions (#1–3) are indicated by open symbols; the three best listeners (#12–14) by filled triangles. The remaining listeners are indicated by gray crosses. The corresponding data of NH listeners (Rennies et al., 2019) are shown in the right panel together with a schematic sketch of the BRIR envelope, which consisted only of direct sound in this experiment (see inset). The asterisk indicates that the NH data were shifted vertically to account for systematic differences between the data sets, see “Stimuli and Conditions.” SRTs = speech recognition thresholds; NH = normal-hearing; BRIR = binaural room impulse responses.

The purpose of Exp. I was to assess listener performance in comparatively simple conditions not involving any reflections. The left panel of Figure 2 shows SRTs measured for HI listeners. Data of NH listeners are shown in the right panel for comparison. Squares and error bars represent the mean values and interindividual standard deviations. Other symbols show individual data, where the three worst HI listeners in the *D*${}_{0}$*N*${}_{0}$ reference condition (#1–3) are highlighted as open symbols, the three best listeners (#12–14) as filled triangles, and the remaining listeners as gray crosses. This code of symbols is the same throughout the article (cf. audiograms in Figure 1 and other experiments below) to track the performance of these listeners through the different experiments. SRTs in the baseline condition (*D*${}_{0}$*N*${}_{0}$) showed that all listeners were well capable of performing the (aided) basic speech recognition task. The mean SRT of −6.7 dB SNR was only marginally higher than in the comparison data of NH listeners (−7.7 dB SNR). This generally good performance of aided listeners with moderate hearing loss in this relatively simple condition is in line with previous studies employing the Oldenburg sentence test (e.g.,  Schädler et al., 2020; Völker et al., 2015). Similar observations were also made by Vermiglio et al. (2012), who found no statistically significant differences in SRTs measured with the American English hearing in noise test between groups of participants with normal audiograms and (unaided) groups with slight, mild, moderate, or severe high-frequency hearing losses. In the baseline condition of the present study, some HI listeners performed better than the NH average. The interindividual variance in performance could not be predicted by PTA${}_{4}$ (as indicated by the coefficient of determination *R*${}^{2}$) or PTA${}_{\mathrm{LF}}$ (*R*${}^{2}$=0.01 for both measures).

The difference in SRTs between HI and NH listeners was larger in the dichotic conditions (about 2.8 dB for the *D*${}_{0}$*N*${}_{\pi }$ condition and 4.0 dB for the *D*${}_{\pi }$*N*${}_{0}$ condition), that is, the average binaural benefit compared to the *D*${}_{0}$*N*${}_{0}$ condition was smaller. However, all HI listeners showed at least a residual binaural benefit, that is, SRTs were always lower in the dichotic conditions than in the diotic baseline condition by at least 1.8 dB. On a group level, SRTs in this experiment differed significantly (*F*[1.724,22.414]=93.679, *p*<.001, $\eta^{2}$=0.878). Post-hoc pairwise comparison with Bonferroni correction showed that SRTs in both dichotic conditions were significantly lower than in the diotic condition (both *p*<.001), and that SRTs in the *D*${}_{\pi }$*N*${}_{0}$ and *D*${}_{0}$*N*${}_{\pi }$ conditions did not differ significantly. The largest binaural advantage among HI listeners (#13, left-pointing triangles) was as large (*D*${}_{\pi }$*N*${}_{0}$) or even larger (*D*${}_{0}$*N*${}_{\pi }$) than the average binaural benefit of NH listeners. There was a significant linear correlation between the pure-tone threshold measures and SRTs in the *D*${}_{\pi }$*N*${}_{0}$-condition (*p*<.001) and a considerable portion of the interindividual SRT variance could be explained by PTA${}_{4}$ (*R*${}^{2}$=0.43) and PTA${}_{\mathrm{LF}}$ (*R*${}^{2}$=0.57), which was expected from results of earlier studies (e.g., Neher, 2017). The best (worst) listeners in the baseline condition generally also had the lowest (highest) SRTs in the binaural conditions, that is, the performance ranking was similar although the interindividual variance was larger (Spearman rank correlations 0.87 and 0.82, respectively). Overall, baseline SRTs predicted 73% (*D*${}_{0}$*N*${}_{\pi }$) and 64% (*D*${}_{\pi }$*N*${}_{0}$) of the variance in the dichotic conditions.

### Experiments II and III: Integration of a Single Reflection

**Figure 3.**
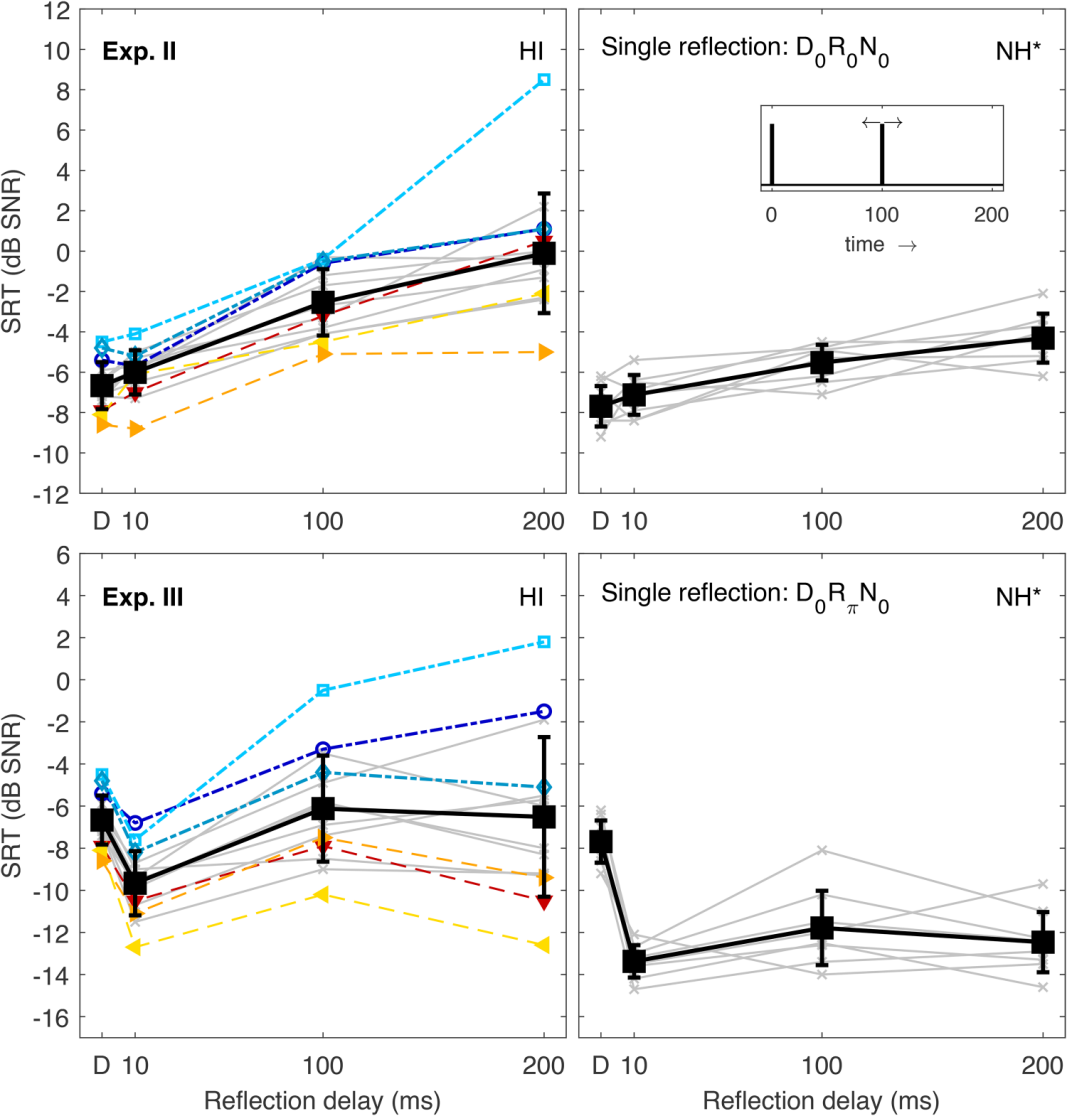
SRTs Measured in Exp. II (top left) and III (bottom left). Individual and mean data are represented as in Figures 1 and 2. The corresponding data of NH listeners (Rennies et al., 2019) are shown in the right panels together with a schematic sketch of the BRIR envelope, which consisted of direct sound and a single reflection with varying delay (see inset). The asterisk indicates that the NH data were shifted vertically to account for systematic differences between the data sets, see “Stimuli and Conditions.” SRTs = speech recognition thresholds; NH = normal-hearing; BRIR = binaural room impulse responses.

Exp. II explored temporal integration of a single speech reflection (same amplitude as the direct sound) in diotic conditions. SRTs for reflection delays between 10 and 200 ms are shown in the top left panel of Figure 3. Symbols and line styles are the same as in the previous figures. Comparison data of NH listeners is again shown in the corresponding right panel. In general, SRTs increased with increasing reflection delay as observed for NH listeners. The ANOVA indicated that the effect of reflection delay was significant (*F*[1.517,20.426]=81.995, *p*<.001, $\eta^{2}$=0.863). Post-hoc comparisons showed that all four SRTs differed significantly from each other (*p*≤.007), suggesting that even a reflection with a short delay of 10 ms produced an increase in SRTs, which had been slightly smaller and not statistically significant in the NH data. Compared to SRTs of NH listeners, the mean SRT increase at the longest delay relative to the baseline condition (*D*) was considerably larger (6.5 dB vs. 3.4 dB), although some HI listeners showed an increase comparable to NH listeners. Other HI listeners had difficulties at the longest delay, some of them requiring positive SNRs to achieve 50% speech intelligibility (up to +8 dB for listener #2). Again, the HI listeners with better (worse) baseline performance tended to perform better (worse) in the condition with a late reflection, as indicated by the symbols in Figure 3, which is confirmed by the significant linear correlation (*p*<.001) and the considerable portion of variance at the longest delay that could be explained by SRT variance in the baseline condition (*R*${}^{2}$=0.56). The individual delay deficit (measured as the SRT difference between the direct sound-only condition and the 200-ms delay condition) was not significantly correlated to any hearing-loss measure derived from the audiograms. Overall, the data of this experiment were in agreement with a previous study (Rennies et al., 2013), which reported an average delay deficit of 7.7 dB for unaided HI listeners (compared to 4.1 dB for NH listeners) for the same types of BRIRs. Note that this comparison suggests that NH listeners are able to focus on one of the BRIR components (ignoring the other), which would produce a 3-dB SRT increase due to the loss in speech energy compared to the direct sound-only condition, whereas some HI listeners experience an additional detrimental effect of the late reflection beyond the loss useful target energy.

Exp. III employed the same reflection delays, but here the reflection had a π-IPD, which had been found to be highly beneficial in NH listeners (cf. Rennies et al., 2019: and bottom right panel of Figure 3). NH SRTs dropped (improved) significantly when a reflection with π-IPD was added to the diotic direct sound and, remarkably, SRTs remained low even when the delay increased to 200 ms (there was no statistically significant difference between delays of 10 and 200 ms). Rennies et al. (2019) argued that NH listeners were able to fully focus on the reflection to extract speech information, independent of its delay. The corresponding SRTs of HI listeners are shown in the bottom left panel. As for NH listeners, the effect of reflection delay on SRTs was significant (*F*[1.454, 18.904]=15.896, *p*<.001, $\eta^{2}$=0.550). In particular, the SRT dropped significantly (*t*[13]=13.674, *p*<.001) when a reflection with a delay of 10 ms was added relative to the baseline condition, that is, there was a benefit due to the additional IPD information. On average, this group benefit at the shortest reflection delay was about half as large as for NH listeners (3.0 dB vs.  5.7 dB). Note that each HI listener showed an SRT improvement at the shortest delay, which is in line with data of Exp. I that indicated that all listeners had at least a residual binaural hearing benefit (Figure 2). However, individual performance differed widely at longer reflection delays: the best-performing HI listeners had SRTs comparable to NH data, that is, their SRTs at the longer delays remained below their baseline SRTs. In contrast, the worst performing listeners had SRTs clearly above baseline, indicating that they failed to benefit from the IPD information at longer reflection delays compared to a condition without IPD information in which all the target energy was confined to the direct sound. On average, SRTs at the two longer delays did not differ significantly from the baseline SRT in this experiment. It should be pointed out, however, that all HI listeners had lower SRTs in Exp. III than in Exp. II at all reflection delays (compare top and bottom left panels of Figure 3, all three comparisons *p*<.001). This means that, even at a delay of 100 or 200 ms where there was no group benefit relative to the baseline condition (in contrast to NH listeners for who a group benefit had been found), the IPD information of the reflection was still advantageous compared to a diotic reflection. This could mean that listeners, who are unable to “ignore” the direct sound and to focus on a late reflection if it contains useful IPD information, can still benefit from the IPD information introduced by the reflection, possibly because it helps them to segregate between the direct sound and the disturbing reflection. As observed before, a considerable portion of the large interindividual variance at the longest delay could be explained by the SRT variance in the baseline condition (*R*${}^{2}$=0.61). A similarly high portion of variance (*R*${}^{2}$=0.55) could be explained by individual IPD benefits in conditions without reflection (*D*${}_{0}$*N*${}_{0}$ vs. *D*${}_{\pi }$*N*${}_{0}$, Figure 2).

### Experiments IV and V: Effects of Reflection Amplitude

**Figure 4.**
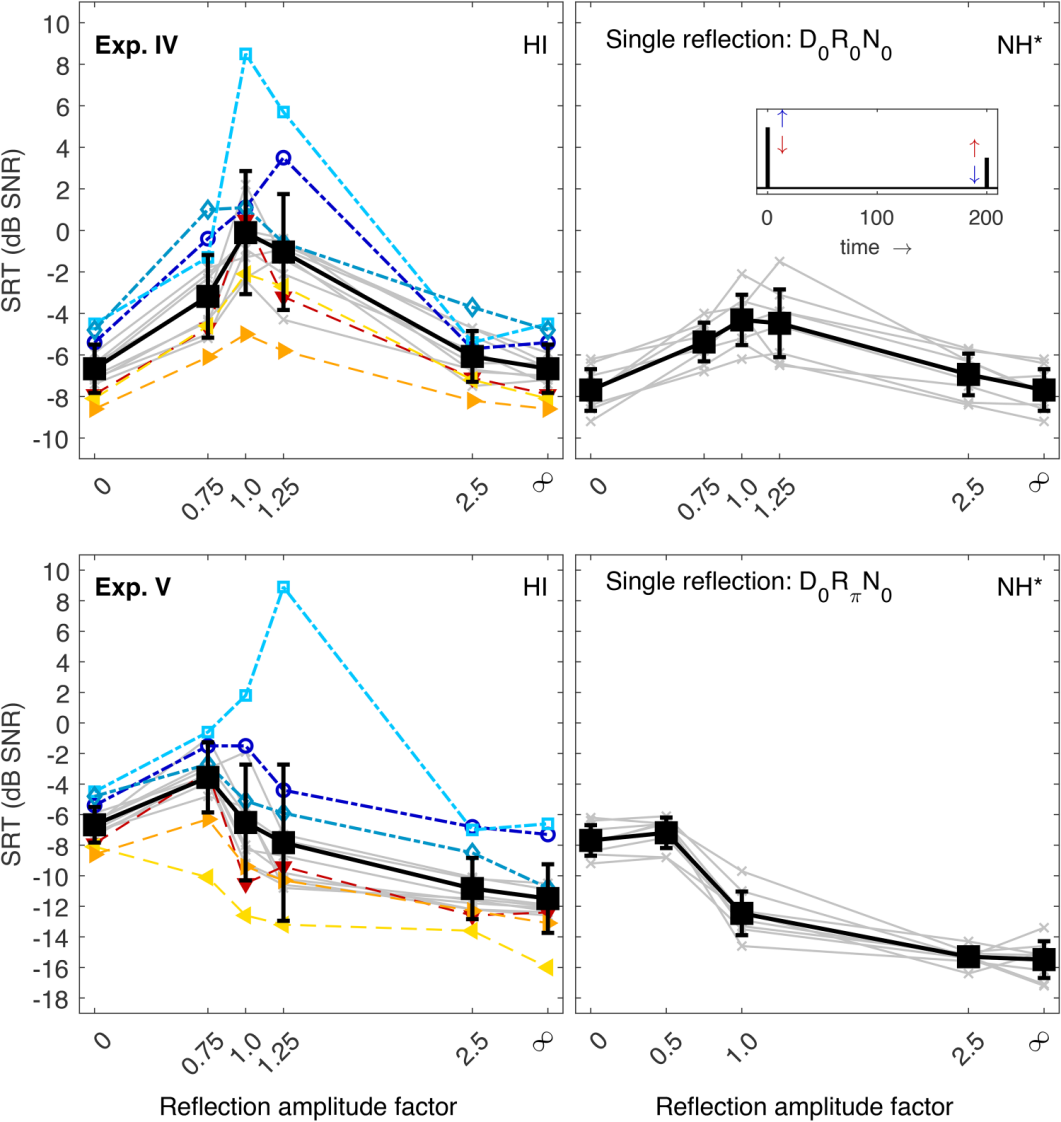
SRTs Measured in Exp. IV (top left) and V (bottom left). Individual and mean data are represented as in the previous figures. The corresponding data of NH listeners (Rennies et al., 2019) are shown in the right panels together with a schematic sketch of the BRIR envelope, which consisted of direct sound and a single reflection with varying relative amplitudes at constant overall speech level (see inset). The asterisk indicates that the NH data were shifted vertically to account for systematic differences between the data sets, see “Stimuli and Conditions.” SRTs = speech recognition thresholds; NH = normal-hearing; BRIR = binaural room impulse responses.

Experiments IV and V explored the role of reflection amplitude for a fixed delay of 200 ms. SRTs as a function of the amplitude amplification factor α are shown in Figure 4. For diotic conditions (Exp. IV, top panels), SRTs increased with increasing reflection amplitude up to α equal to 1 (equally strong direct sound and amplitude), and then decreased. Note that the rightmost data points (α= ∞) in Figure 4 were taken here as a copy from leftmost data points (α=0) to illustrate the expected symmetry: The overall energy of the speech signal was held constant as described above and, consequently,“α= ∞” means that there was no direct sound but only the delayed reflection, which now had the role of a delayed direct sound. In other words, if the normalized BRIR consists only of a single diotic component (direct sound or reflection), SRTs are expected to be the same because they only differ in an onset delay of the target speech. The main effect of α was significant (*F*[1.816,23.603]=59.472, *p*<.001, $\eta^{2}$=0.821). Post-hoc tests showed that SRTs did not differ for the two extreme α values (0 and 2.5), whereas these two SRTs were significantly lower than for intermediate values of α (all *p*<.001). The SRT for α equal to 0.75 was also significantly lower than for values of 1 and 1.25 (*p*≤.002). In comparison to NH data, the SRT increase at values of α close to 1 was more pronounced in HI listeners and exceeded the 3-dB increase which would be expected if listeners could just ignore the reflection as discussed above. This could be expected based on the results of Exp. II (Figure 3). Those listeners who struggled most due to the reflection (positive SRTs in the mid-α range) had also performed relatively poorly in the previous experiments. Note, however, that all listeners had SRTs very similar to their reference SRTs for α equal to 2.5 (as confirmed by the insignificant differences on a group level). In other words, a late reflection, which is detrimental for some listeners when it has a similar amplitude as the direct sound, is useful when its amplitude is larger than that of the direct sound. This could mean that all HI listeners were able to use the intensity advantage of the late reflection to focus on the reflection rather than on the direct sound, while they were not all able to exploit an IPD advantage to focus on the late reflection, when the reflection amplitude was the same as of the direct sound (see Exp. II above).

SRTs resulting from the same variations in amplification factor, but with a π-IPD reflection (Exp. V) are shown in the bottom panels of Figure 4. In this representation, the rightmost data points (α= ∞) were taken from the *D*${}_{\pi }$*N*${}_{0}$ condition of Exp. I, again assuming that a BRIR with a single component should produce the same SRTs in stationary noise irrespective of a difference in target speech onset. The average SRT pattern was generally similar to the NH data in that SRTs decreased relative to the baseline condition (α=0) for amplification factors larger than 1 (note that not exactly the same α values had been measured with NH listeners by Rennies et al., 2019). While the average SRT for α equal to 1.25 did not differ significantly from α equal to 0 (probably at least in part due to the one strong outlier, listener #2, see open squares), the SRT was significantly lower for α equal to 2.5 (*p*<.001). For this amplification factor, the SRT was still significantly higher than for the *D*${}_{\pi }$*N*${}_{0}$ condition (α=∞, *p*= .009), although the difference was small (about 0.7 dB). This means that HI listeners seemed to be able to exploit the IPD advantage of the late reflection when its energy was sufficiently high relative to the direct sound.

### Experiments VI and VII: Integration of an Increasing Number of Early Reflections

Experiments VI to IX explored the HI listeners’ capability to integrate more than one reflection. In these experiments, the reflections always had the same amplitude as the direct sound, and the level of all components was adjusted so that the overall level was always the same independent of the number of BRIR components. During these experiments, some listeners were not able to complete the SRT measurement for higher numbers of reflections. In other words, they consistently failed to correctly repeat more than two out of the five words of a sentence even at an SNR of +20 dB, which caused the adaptive procedure to abort the track to avoid too high speech levels of more than 85 dB SPL plus individual NAL-RP amplification. Because of these huge interindividual differences, the subsequent experiments were not statistically analyzed on a group level. Instead, the conditions provoking these differences are discussed in detail for the different listeners.

**Figure 5.**
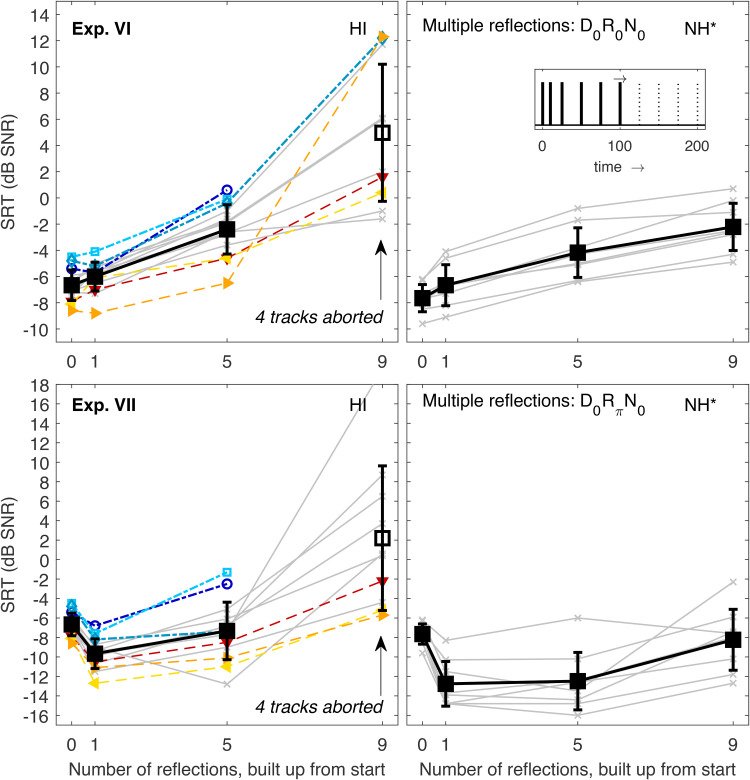
SRTs Measured in Exp. VI (top left) and VII (bottom left). Individual and mean data are represented as in the previous figures with the exception that the mean SRTs could not be measured for all listeners in the most difficult conditions. Unfilled black squares indicate mean values that were computed across a smaller number of listeners. These conditions are also marked by labelled arrows indicating the number of unmeasurable tracks. The corresponding data of NH listeners (Rennies et al., 2019) are shown in the right panels together with a schematic sketch of the BRIR envelope, which consisted of direct sound and multiple reflections built up from the front (see inset). The asterisk indicates that the NH data were shifted vertically to account for systematic differences between the data sets, see “Stimuli and Conditions.” SRTs = speech recognition thresholds; NH = normal-hearing; BRIR = binaural room impulse responses.

In Exp. VI, diotic stimuli were used and an increasing number of reflections (starting with the lowest delay) was added to the direct sound (thereby decreasing the relative energy of each BRIR component). SRTs are shown in the top panels of Figure 5. For one reflection (delay 10 ms) and five reflections (i.e., with a maximum reflection delay of 100 ms, see Table 2), SRTs of HI listeners monotonically increased as observed for NH listeners. The average increase for five reflections relative to the direct sound-only condition was only slightly larger (4.3 dB vs.  3.5 dB), and the SRT of the worst HI listener (0.6 dB) was only slightly higher than the SRT of the worst NH listener (−0.8 dB). However, when the BRIR consisted of nine reflections with a maximum delay of 200 ms, SRTs increased considerably for most HI listeners. Four listeners (#1, 2, 4, and 6) were not able to complete the task as indicated by the arrow in the panel. The unfilled black square represents the average SRT across the remaining 10 listeners. Even without the four worst listeners with unmeasurable SRTs, the average was more than 7 dB above the NH average, and the worst (measurable) SRT was more than 11 dB higher than the worst NH listener. In contrast, some HI listeners showed normal or close-to-normal performance in this condition, that is, their SRTs increased only slightly from five to nine reflections. Interestingly, the SRT of listener #14 (whose SRTs were among the best in all previous experiments) also increased dramatically at the highest number of reflections. It is unclear whether this is a single outlier possibly caused by lapses of attention or if this represents a condition where even this otherwise well-performing HI listener had trouble recognizing the target speech.

Exp. VII (bottom panels of Figure 5) used the same BRIRs, but here reflections had a π-IPD. In general, SRTs of HI listeners showed a similar pattern as in Exp. VI in that all listeners could perform the task up to five reflections, but not for nine reflections. For five reflections, eight HI listeners had lower SRTs than in the direct sound-only condition, suggesting that they could exploit the IPD information even when it was spread across multiple reflections (as observed for NH listeners). For six listeners, the SRT for five reflections was the same or higher than for direct sound-only, indicating that the IPD-advantage was outweighed by the fact that it was spread across multiple reflections. For nine reflections, again four HI listeners (#1, 2, 3, and 9) could not perform the task. One further listener (#4) had an estimated SRT close to +20 dB, indicating that the adaptive procedure was close to its abortion criterion, and the SRT estimate could be unreliable and at the brink of unmeasurable. In contrast, some other HI listeners had SRTs close to the direct sound-only condition as observed for the NH listeners.

### Experiments VIII and IX: Integration of an Increasing Number of Late Reflections

**Figure 6.**
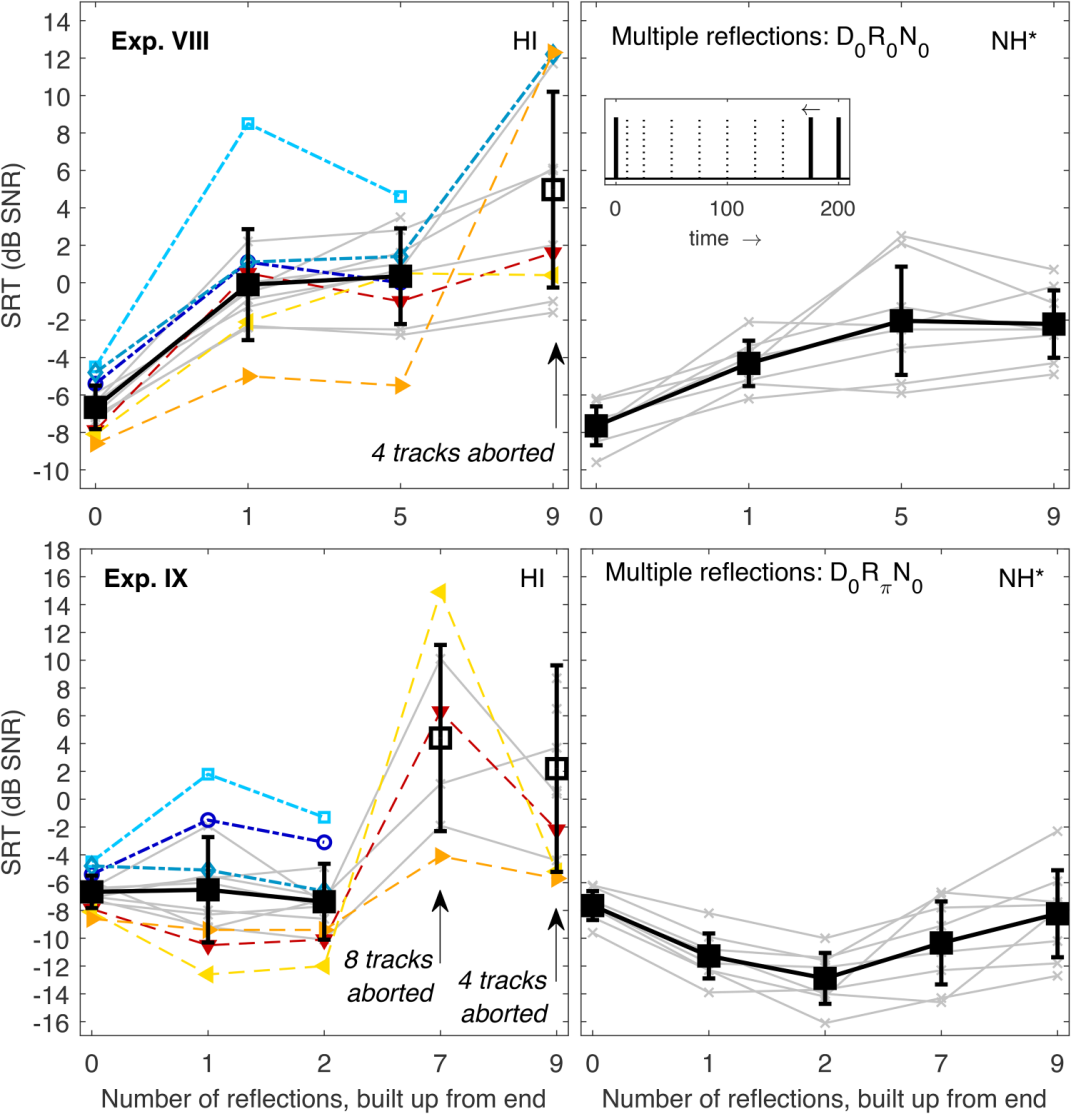
SRTs Measured in Exp. VIII (top left) and IX (bottom left). Individual and mean data are represented as in the previous figures. Data points for which SRTs could not be measured for all listeners are indicated by arrows. The corresponding data of NH listeners (Rennies et al., 2019) are shown in the right panels together with a schematic sketch of the BRIR envelope, which consisted of direct sound and multiple reflections built up from the end (see inset). The asterisk indicates that the NH data were shifted vertically to account for systematic differences between the data sets, see “Stimuli and conditions.” SRTs = speech recognition thresholds; NH = normal-hearing; BRIR = binaural room impulse responses.

The final two experiments also employed multiple reflections spread across the same 200-ms window, but here the reflections were gradually added starting with the longest delay. SRTs for diotic stimuli (Exp. VIII) are shown in the top panels of Figure 6. Note that the last data point (nine reflections) is the same as the last data point of Exp. VI because in both cases all nine reflections were included. Similarly, the data points for one reflection were taken from Exp. 2 (Figure 3, top left, last data points). In general, the trend from direct sound-only to one and five reflections was similar to the monotonic SRT increase observed for NH, with a slightly stronger increase for HI listeners (7.0 dB vs. 5.6 dB). The data for nine reflections were already discussed above: where NH listeners had a moderate further SRT increase from five to nine reflections, some HI listeners struggled heavily, and four SRTs could not be measured at all (#1, 2, 4, and 6).

Data of Exp. IX are shown in the bottom panels of Figure 6. Reflections were also gradually added starting from the longest delay, but here the reflections had a π-IPD. The NH data showed a V-shaped trend: SRTs decreased when a reflection with useful IPD-information was added (delay 200 ms), and further decreased when another reflection (delay 175 ms) was added, because the overall energy of BRIR components with IPD-advantage was larger. Increasing the number of reflections to seven and nine caused SRTs to rise again, presumably because the BRIR components were spread over too large a temporal window to be fully integrated (Rennies et al., 2019). For 0, 1, and 2 reflections, some HI listeners (the generally better ones, see triangles) showed a trend similar to NH listeners. On a group level, however, SRTs were basically unchanged because other HI listeners could not benefit from the IPD-advantage of the late reflections in the same way and some even showed an SRT increase (cf. Exp. III).

When the number of reflections was increased to seven (i.e., when five more reflections with shorter delays down to 50 ms were added), many HI listeners had difficulties performing the task: for eight out of 14 listeners no SRTs could be measured (#1–4, 6, and 9–11). Even listeners who had shown very good SRTs in the other experiments and in the conditions with fewer reflections in this experiment had very strong SRT increases for seven reflections. For example, listeners #12 and 13 had SRTs that were 16.4 and 26.9 dB higher, respectively, for seven reflections than for two reflections (see downwards- and left-pointing triangles). The best HI listener (#14, right-pointing triangles) had an increase of 5.3 dB, while the average increase for NH listeners was 2.6 dB. Interestingly, SRTs improved for most HI listeners when two more reflections with short delays (10 and 25 ms) were added. Here, “only” four listeners could not perform the task (#1, 2, 3, and 9). For the two “otherwise good” listeners whose SRTs had increased strongly from two to seven reflections (downwards- and left-pointing triangles), SRTs dropped considerably by 20.1 and 8.5 dB, respectively.

It appears that the condition with seven reflections was particularly difficult for many HI listeners. This conditions consisted of the diotic direct sound and seven reflections with π-IPD with delays between 50 and 200 ms. The comparison to conditions with only one or two reflections as well as to Exp. III (Figure 3, bottom) suggests that it is not the reflection delay *per se* that caused major problems, because all listeners could measure SRTs for a single reflection delayed by 100 or 200 ms, and for two late reflections delayed by 200 and 175 ms. Moreover, SRTs were generally better with nine than with seven reflections, so it is not the number of reflections *per se* either. It seems that a range of temporal gaps between the direct sound and the first reflection around 50 ms (for seven reflections) makes the task abnormally difficult for HI listeners. Conceptually, these observations could be explained by assuming that some HI listeners could focus on the late reflections as long they were sufficiently separable from the direct sound. This would explain decreasing SRTs for one and two reflections as observed for some HI listeners (and for NH listeners). As the temporal gap between direct sound and reflections decreased, “ignoring” the direct sound could have become more difficult up to the point of making SRT measurements impossible. Alternatively, some HI subjects may have always focused on the direct sound “ignoring” the late reflections with IPD-advantage. This would explain an increase in SRTs by about 3, 5, and 9.5 dB for one, two, and seven reflections, respectively (cf. Table 2 in Rennies et al., 2019), which was found for some HI listeners at least for a single reflection. These listeners, too, could have been disturbed in their strategy by reflections with different IPDs that were temporally too close to the direct sound. It would be interesting to investigate these effects further in future experiments with intermediate numbers of reflections (between seven and two) and/or intermediate temporal gaps between direct sound and first reflection (between 50 and 175 ms) to explore the point at which HI listeners start experiencing difficulties.

Clearly, NH listeners did not show any sign of comparable difficulties. Their behavior was consistent with the assumption that they were able to focus on the reflections which carried an IPD-advantage in all conditions. If they had always focused on the direct sound, their SRTs would have been higher by about 10 dB for nine reflections than for direct sound-only because of the reduced relative energetic contribution of the direct sound in the normalized BRIR (cf. Table 2 in Rennies et al., 2019). In fact, SRTs were slightly lower for nine reflections than for direct sound only, suggesting a small binaural benefit. This benefit was much smaller than for *D*${}_{\pi }$*N*${}_{0}$ because the reflections were spread over a large temporal distance and could not be fully integrated.

## General Discussion and Conclusions

With respect to the research questions outlined in the introduction, the following conclusions can be drawn from the data:

A: The present group of HI listeners can make use of early reflections similarly to NH listeners, but a larger spread in performance is generally observed.
B: The same HI listeners are in general less able to “ignore” the direct sound and to optimally “shift” their temporal integration window than NH listeners.
C: Some HI listeners show a remarkable inability to integrate across multiple reflections, which is quite dissimilar to NH listeners. This effect is most pronounced in conditions requiring spatial and temporal integration.
D: Only some of the observed interindividual variability observed in the present group of HI listeners can be well predicted by audiogram-derived measures. In contrast, speech performance in less complex conditions was often found to be highly correlated to performance in complex conditions.

The present group of HI listeners had normal or close-to-normal aided SRTs in the *D*${}_{0}$*N*${}_{0}$ reference condition (Exp. I), indicating that they were well able to recognize speech in a simple condition without temporal integration of reflections or binaural processing. The HI listeners were also able to process binaural information, at least to a limited degree, because all had lower SRTs in simple binaural conditions (*D*${}_{\pi }$*N*${}_{0}$ or *D*${}_{0}$*N*${}_{\pi }$, see Exp. I). Furthermore, they could generally cope with a single (even very late) reflection in diotic conditions (Exp. II), although interindividual variability was larger for long delays. Thus, in relatively simple listening conditions, most of our primarily older HI listeners performed similarly to young NH listeners (the age range was 18–31 years in the study of Rennies et al., 2019). Data from experiments with more complex spatio-temporal integration, however, clearly showed that this did not automatically imply that they also had close-to-normal performance in more complex conditions. For example, the (residual) capability to use IPD-information in simple conditions did not imply that all HI listeners could exploit IPD-information carried by a late reflection in the way NH listeners could (Exp. II). In conditions with a single late reflection (experiments II to V) the SRT ranking of the listeners appeared to be quite consistent, and a significant part of the interindividual variance could be explained by SRTs in the *D*${}_{0}$*N*${}_{0}$ reference condition. This suggests that—in the present group of listeners—some listeners had “relatively good” and others “relatively poor” performance across conditions. Simple measures derived from the audiogram (PTA${}_{4}$ and PTA${}_{\mathrm{LF}}$) predicted only a smaller portion of the variance than SRTs in the baseline condition, which may be expected because reduced audibility was partly compensated for by the prescriptive amplification. It should be noted that this may be in part due to the relatively small sample size tested in this study. It is possible that a clearer relation between hearing loss and SRTs could be found if a larger sample size was measured or if age or etiologies of hearing loss would have been better controlled. A rather consistent ranking was also observed in conditions with varying reflection amplitude (experiments IV and V). These data also showed that all HI listeners returned to close-to-normal performance when the reflection energy was considerably higher than the energy of the direct sound. Energetic cues were thus helpful to overcome listening difficulties with a late reflection.

The observation that some subjects performed generally better than others across different conditions was also reported for speech recognition tasks with more complex maskers than the stationary noise employed here. For example, Kidd et al. (2019) measured SRTs of a target talker in the presence of two interfering talkers. They manipulated the target-to-masker similarity/dissimilarity by introducing sex differences between target and maskers, spatial separation between target and maskers, and time-reversal of the maskers (making them unintelligible). Using the same audibility-loss compensation as in the present study (linear NAL-RP prescription), Kidd et al. (2019) found considerable interindividual differences in SRTs in all conditions. Interestingly, the ranking of individuals across conditions was very similar (see their Figure 7), even though exploiting the different unmasking cues likely relies on very different processing strategies like identifying differences in fundamental frequency and vocal tract length (for sex differences), binaural processing (for spatial separation), and negation of masker intelligibility (for time-reversed maskers). Kidd et al. (2019) hence argued that the performance of the relatively poor subjects “appears to be a reflection of a more general problem affecting multiple abilities rather than being strongly related to reduced resolution of any specific cue per se.” This is in line with the notion that no specific and distinct binaural impairment factor can be singled out—as discussed in the introduction—but instead a general suprathreshold processing deficit causes variations across hearing-impaired subjects, such as the “D-component” (for “distortion”) proposed by Plomp (1978). Such a “D-component” was employed in recent modelling approaches by Kollmeier et al. (2016) and Hülsmeier et al. (2021) as an individual jitter of the internal stimulus representation, which could be interpreted as an individual factor to describe the capacity to process the available information (beyond what is lost by limited or only partly restored audibility). Again, it should be emphasized that the present sample size is too small to draw conclusions about the general HI population, especially since many individual traits and basic psychoacoustic performance metrics were not measured. Running prediction models and systematically varying different types of processing deficits (such as audibility, binaural processing, or the “D-component”) could help to better understand the reasons underlying the observed interindividual variability. Likewise, extensions of the present study with NH and HI listener groups matched in age and/or cognitive performance could shed more light on the role and interactions of age, cognitive factors, and increased hearing thresholds in speech intelligibility in spatio-temporal integration tasks. Similarly, the role of audibility should be investigated in more detail, e.g., by also measuring SRTs in unaided HI listeners or by employing different amplification schemes. Nevertheless, despite the small sample size and the unknown hearing loss etiologies the results of the present study suggest that a consistent performance ranking of individual HI listeners across conditions can also be observed for less complex maskers without any impact of “informational masking” (IM). IM occurs when interferers share perceptual attributes with the target that can draw attention away from the target, lead to explicit confusion of masker and target words, or generally cause uncertainty in the observer (e.g., see review in Kidd & Colburn, 2017). Based on the concept of “generally good/bad” subjects, one could speculate that subjects who struggle with, for example, spatio-temporal integration of reflections would also be poor performers in speech-on-speech masking conditions. However, this remains to be shown in future studies.

The largest performance differences between listeners in the present study were observed in conditions with multiple reflections, especially when the BRIR components spread over a period of 200 ms. In these conditions, some HI listeners failed to reach 50% speech recognition at an SNR of +20 dB (at which point the employed adaptive procedure aborted the track). Listeners with unmeasurable SRTs were often the same across the different experiments and conditions, and most frequently included listeners #1, 2, and 3, that is, the listeners with the worst SRTs in the baseline condition. However, the relatively consistent ranking of HI listeners was not as obvious as in conditions with fewer reflections. For example, one of the generally best performers had the worst (measurable) SRT in Exp. VIII. While this may have been a random outlier, this seems an unlikely explanation for the data measured in Exp. IX: Here, SRTs could not be measured for more than half of the HI listeners in one condition. Remarkably, this condition also revealed that otherwise very good HI listeners had considerably increased SRTs, while they had shown normal SRTs in all other conditions of the present study. Further research is needed to investigate in more depth what makes this condition stand out, for example, if a particular combination of stimulus parameters can be identified to measure a threshold at which major difficulties arise even for generally good listeners. Furthermore, these data may be a challenge for the majority of existing models which do not include a specific binaural impairment factor, but instead focus on audibility as the main driver of reduced performance (see section “Introduction”) or a more general suprathreshold deficit factor. It seems unlikely that such models could explain the specific difficulties of some listeners which arise in conditions with multiple reflections, especially when diotic direct sound was combined with π-IPD reflections (see Figure 6).

From a clinical perspective, it would be interesting to compare the performance in conditions like the ones tested here with basic psychoacoustic performance measures, measures of cognitive function, aspects such as hearing-aid satisfaction in aided real-life listening conditions, or more generally to include such conditions in auditory profiling (see, e.g., Sanchez-Lopez et al., 2020; Iliadou et al., 2022; Saak et al., 2022). It is possible that a systematic manipulation of spatio-temporal integration demands in laboratory settings can help to magnify interindividual performance differences and hence to identify listeners with specific difficulties in their everyday life, thus supporting the diagnostics and fitting processes of hearing devices.

Data will be made available upon request.
